# Subgroup variations in effectiveness of femoropopliteal sirolimus versus paclitaxel-coated balloon angioplasty: post hoc results of the randomised SIRONA trial

**DOI:** 10.1186/s42155-026-00743-2

**Published:** 2026-08-11

**Authors:** Ulf Teichgräber, Maja Ingwersen, Thomas Lehmann, Stephanie Platzer, Ina Kunstmann, Sabine Steiner, Andrej Schmidt, Tim Wittig, Thomas Zeller, Marcus Thieme, Hans Krankenberg, Martin Werner, Marianne Brodmann, Nasser Malyar, Stefan Müller-Hülsbeck, Luca de Paoli, Oliver Schlager, Martin Austermann, Gunnar Tepe, Christian Erbel, Wulf Ito, Rainer Waßmer, Lars Maiwald, René Aschenbach, Christian Wissgott, Martin Andrassy, Florian Wolf, Ulrich Beschorner, Maciej Pech, Konstantinos Katsanos, Dierk Scheinert

**Affiliations:** 1 Department of Radiology, Jena University Hospital, Friedrich Schiller University Jena, Jena, Germany; 2 Center for Clinical Studies, Jena University Hospital, Friedrich Schiller University Jena, Jena, Germany; 3 Department of Angiology, University Hospital for Internal Medicine II, Medical University Vienna, Vienna, Austria; 4 Department of Angiology, University of Leipzig Medical Center, Leipzig, Germany; 5 Department of Angiology, University Heart Center Bad Krozingen, University Hospital Freiburg, Bad Krozingen, Germany; 6 Department of Vascular Medicine, Sana-Kliniken Oberfranken Coburg GmbH, Coburg, Germany; 7 Department of Angiology, Hanusch Hospital Vienna, Vienna, Austria; 8 Division of Angiology, Medical University Graz, Graz, Austria; 9 Department of Cardiovascular Medicine, University of Münster, University Hospital Münster, Münster, Germany; 10 Department of Radiology, DIAKO Hospital Flensburg, Flensburg, Germany; 11 Department of Radiology, KABEG Klinikum Klagenfurt Am Wörthersee, Klagenfurt, Austria; 12 Department of Vascular Surgery, St. Franziskus Hospital Münster, Münster, Germany; 13 Department of Radiology, RoMed Klinikum Rosenheim, Rosenheim, Germany; 14 Department of Cardiology and Angiology, University Hospital Heidelberg, Heidelberg, Germany; 15 Department of Angiology, Cardiovascular Center Klinikverbund Allgäu, Immenstadt, Germany; 16 Department of Angiology, Elblandklinikum Radebeul, Radebeul, Germany; 17 Department of Angiology, Kreiskrankenhaus Torgau, Torgau, Germany; 18 Department of Radiology, Schön Clinic Rendsburg, Rendsburg, Germany; 19 Department of Cardiology, Fürst-Stirum-Klinik Bruchsal, Bruchsal, Germany; 20 Department of Radiology, University Hospital for Radiology, Medical University Vienna, Vienna, Austria; 21 CoreLab Black Forest, Bad Krozingen, Germany; 22 Department of Radiology and Nuclear Medicine, University Hospital Magdeburg, Otto Von Göricke University, Magdeburg, Germany; 23 Department of Radiology, Patras University Hospital, Patras, Greece; 24 Department of Radiology, Friedrich-Schiller University Jena, Jena University Hospital, Kastanienstraße1, Jena, 07747 Germany

**Keywords:** Angioplasty, Paclitaxel, Peripheral artery disease, Sirolimus

## Abstract

**Introduction:**

The randomised SIRONA trial showed that sirolimus-coated balloon (SCB) angioplasty was non-inferior to paclitaxel-coated balloon angioplasty (PCB) regarding femoropopliteal primary patency. There was no difference in clinically driven target lesion revascularisation (cdTLR) rates between the treatment groups. This post-hoc analysis aimed to assess whether the overall results were consistent across different subgroups and selected post-baseline factors.

**Methods:**

Primary patency at 12 months was assessed using duplex ultrasound and adjudicated by a core laboratory for 203 and 199 participants in the SCB and PCB groups, respectively (available-case intention-to-treat analysis). Data on 12-month cdTLR were available for 238 and 244 participants in the SCB and PCB groups, respectively. Odds ratios (ORs) for primary patency and hazard ratios (HRs) for cdTLR after SCB compared to PCB in non-pre-specified subgroups were assessed using generalised linear mixed models to control for centre effects, and Cox proportional hazards models, respectively.

**Results:**

Primary patency and cdTLR were generally consistent across the analysed subgroups. Two nominal interaction signals were observed. For cdTLR, a nominal interaction with age was identified (interaction *p* = 0.047); among participants older than 70 years, the estimated hazard ratio favoured SCB (HR 0.52, 95% CI 0.16–1.71). For primary patency, treatment effects differed according to residual diameter stenosis (interaction *p* = 0.022); the estimated treatment effect favoured SCB in participants with residual diameter stenosis > 30% (OR 1.67, 95% CI 0.76–3.65). No other interactions between treatment and subgroup or post-baseline factors were observed.

**Conclusion:**

The effectiveness of SCB compared to PCB was similar and mainly consistent among subgroups. The evaluation should be considered exploratory.

**Trial registration:**

ClinicalTrials.gov, NCT04475783. Registered 17 July 2020, https://clinicaltrials.gov/study/NCT4475783.

## Introduction

Femoropopliteal artery disease can effectively be treated with paclitaxel-coated balloon (PCB) angioplasty [1], and the 2024 ESC guidelines recommend drug-eluting treatment as the preferred initial interventional strategy [2]. The randomised SIRONA trial recently demonstrated that sirolimus-coated balloon (SCB) was non-inferior to PCB angioplasty regarding primary patency and the composite endpoint of target vessel revascularisation (TVR), major target limb amputation, and device- or procedure related death at 12 months in patients with symptomatic femoropopliteal artery disease. Most patients had intermittent claudication. However, the result for primary patency was close and was not robust in sensitivity analyses [3]. This raises the question of whether the overall results can be applied consistently to the relevant subgroups.

Initial studies on peripheral arteries showed favourable clinical long-term efficacy of SCB [4–6]. However, in patients with coronary artery disease (CAD), it was demonstrated that limus drug-coated balloons resulted in similar clinical, but poorer angiographic outcomes, than PCB [7]. Sirolimus is less lipophilic than paclitaxel, and its bioavailability depends strongly on balloon coating technology; however, its transmural diffusion gradient is higher and it distributes more evenly within the vessel wall due to its more specific binding properties [8]. The immunosuppressive and antimigratory effects of sirolimus are considered to be stronger than those of paclitaxel, at least when applied continuously to the coronary arteries [9]. Furthermore, it has been suggested that sirolimus can inhibit the proliferation of hypoxic cells [10]. Finally, sirolimus does not act through cytotoxicity including apoptosis [9–11]. These pharmacological properties may result in different outcomes depending on lesion characteristics.

To account for the heterogeneity of lesions, we performed a post hoc subgroup analysis of 12-month primary patency and its associated outcome, clinically driven target lesion revascularisation (cdTLR). This evaluation may indicate the need for further investigation to establish which drug is most appropriate for preventing restenosis in specific lesions.

## Methods

### Design of the SIRONA trial

The design and 12-month results of the randomised non-inferiority SIRONA trial have been published [3, 12]. Briefly, patients aged ≥ 18 years with Rutherford category 2–4 symptoms due to single femoropopliteal lesions successfully predilated without flow-limiting dissection were randomised to receive either SCB or PCB at a ratio of 1:1. The study device for SCB angioplasty was the Magic Touch®PTA sirolimus-coated balloon catheter (Concept Medical Inc., Tampa, USA), whereas the control devices were PCBs that had evidence of 2-year efficacy from randomised controlled trials. All study devices were CE-marked and commercially available. Patients were enrolled at 25 centres in Germany and Austria between April 2021 and September 2022. A total of 238 patients were assigned to the SCB group (238 received SCB) and 244 to the PCB group (239 received PCB, 5 received SCB). The study is ongoing with scheduled yearly follow-ups up to 5 years.

Primary patency was defined as the absence of target lesion restenosis as assessed by duplex ultrasonography peak systolic velocity ratio > 2.4 or total occlusion and adjudicated by a core laboratory (CoreLab Black Forest GmbH, Bad Krozingen, Germany) without the need for repeat TLR. All primary vessel patency findings that were assessed as borderline by the core laboratory were adjudicated by a blinded clinical events committee. Participants and outcome assessors were blinded to the treatment allocation, whereas investigators and treating physicians were not.

### Results of the main SIRONA trial

No significant differences were found in overall baseline demographics or clinical characteristics among the treatment groups. The mean age was 68.0 years (standard deviation [SD] 8.9 years), and 311 participants (64.5%) were male. The mean lesion length was 8.4 cm (SD 6.1 cm). Of these lesions, 160 (33.2%) were chronic total occlusions (CTOs) and 125 (28.3%) were classified as heavily calcified by the core laboratory. The majority of participants (96.3%) had intermittent claudication categorised as Rutherford class 2 or 3. The SIRONA trial demonstrated that 12-month primary patency with SCB was non-inferior to PCB (73.9% [150/203] participants and 74.9% [149/199] participants, respectively; absolute risk difference − 1.0% [95% CI − 9.6 to 7.6]). With a 10% non-inferiority margin, the *p*-value for non-inferiority was 0.019. However, sensitivity analyses, considering both missing data and treatment received, did not confirm non-inferiority. There was no difference in cdTLR between the two groups (Kaplan–Meier estimate at 12 months: SCB 93.1% vs. PCB 95.6%: log-rank *p* = 0.58).

### Statistical analysis

Subgroup and post-baseline factor analyses were conducted regarding primary patency using generalised linear mixed models. “Treatment” was the fixed factor, and “centres” the random factor. These models were performed using Jamovi software based on R syntax (The Jamovi Project, 2025, version 2.6.44, retrieved from https://www.jamovi.org). The treatment effects on primary patency are presented as odds ratios (OR) with 95% Wald confidence intervals. Subgroup and post-baseline factor analyses of cdTLR were conducted using Cox proportional hazard models. The likelihood ratio test was used to assess *p*-values for interaction. The **t**reatment effects regarding cdTLR were presented as hazard ratios (HR) with 95% Wald confidence intervals. As the interaction assessment was not pre-specified and no pre-randomisation stratification was performed, and as the number of cdTLR events was small, the results should be considered exploratory. Therefore, we did not set a limit on the interaction *p*-value to define statistical significance. To assess heterogeneity of baseline and procedural characteristics by age and by residual diameter stenosis, respectively, we used chi-squared test (with Yates’s correction for continuity, where applicable), and Fisher’s exact test for categorical variables, and a *t*-test or Mann–Whitney *U* test for continuous variables. *p*-values < 0.05 from two-sided tests were considered to indicate statistical significance. Analyses were performed based on the available-case intention-to-treat study population (XLSTAT, version 2015.6.01.24026, Addinsoft, Paris, France).

## Results

**Fig. 1:**
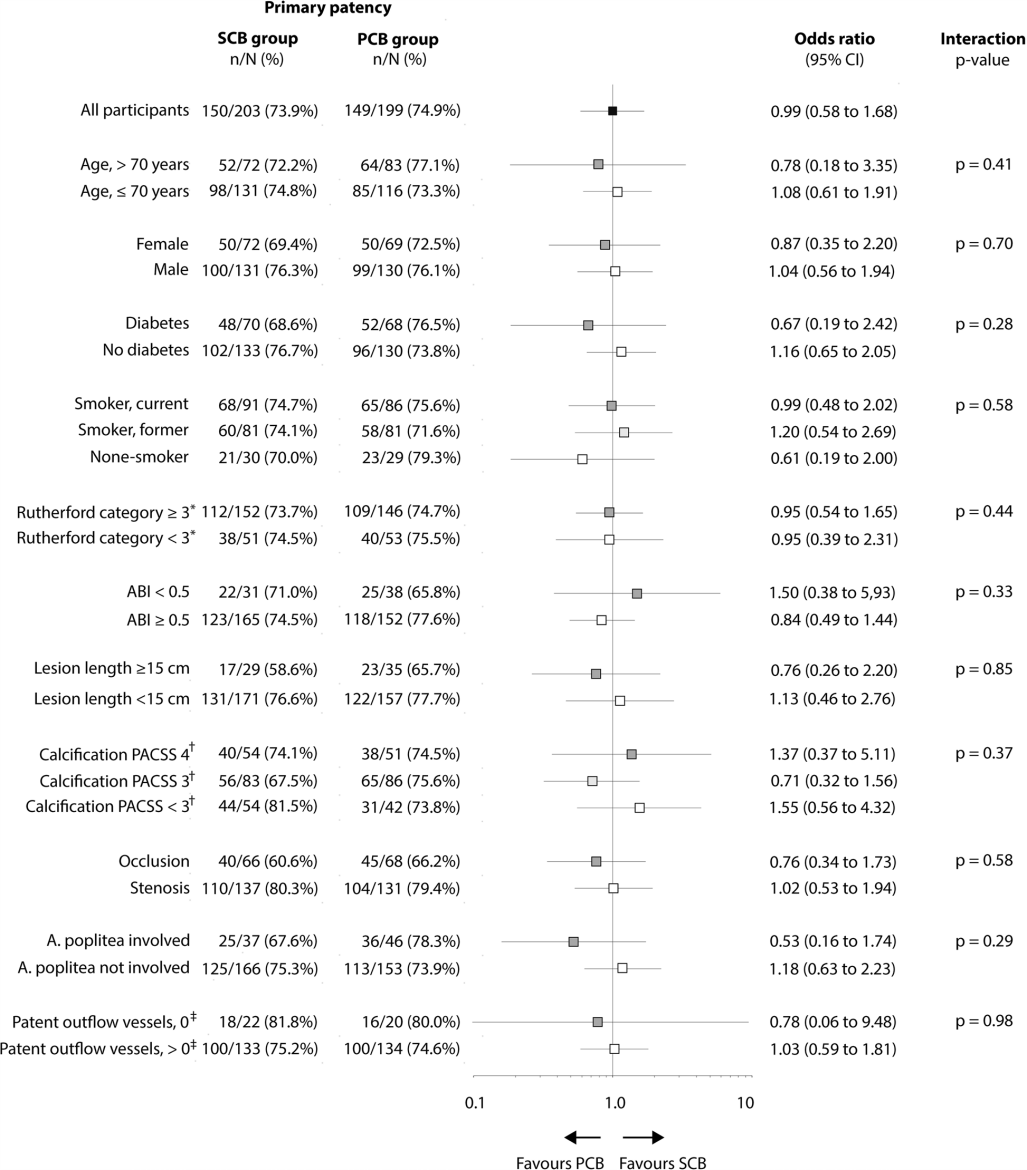
Subgroup analysis of the treatment effect of SCB versus PCB regarding primary patency at 12 months, controlling for centre effects. *Rutherford category 3 denotes moderate claudication. † Chronic limb-threatening ischaemia (Rutherford category > 3) occurred only in a few participants (7 (2.9%) in the SCB group and 10 (4.1%) in the PCB group). ‡ PACSS grade 3 denotes bilateral calcification < 5 cm and PACSS grade 4 bilateral calcification ≥ 5 cm. § Patency of outflow vessels was defined as < 50% diameter stenosis. ABI = ankle-brachial index; PACSS = peripheral arterial calcium scoring system; PCB = paclitaxel-coated balloon; SCB = sirolimus-coated balloon

**Fig. 2:**
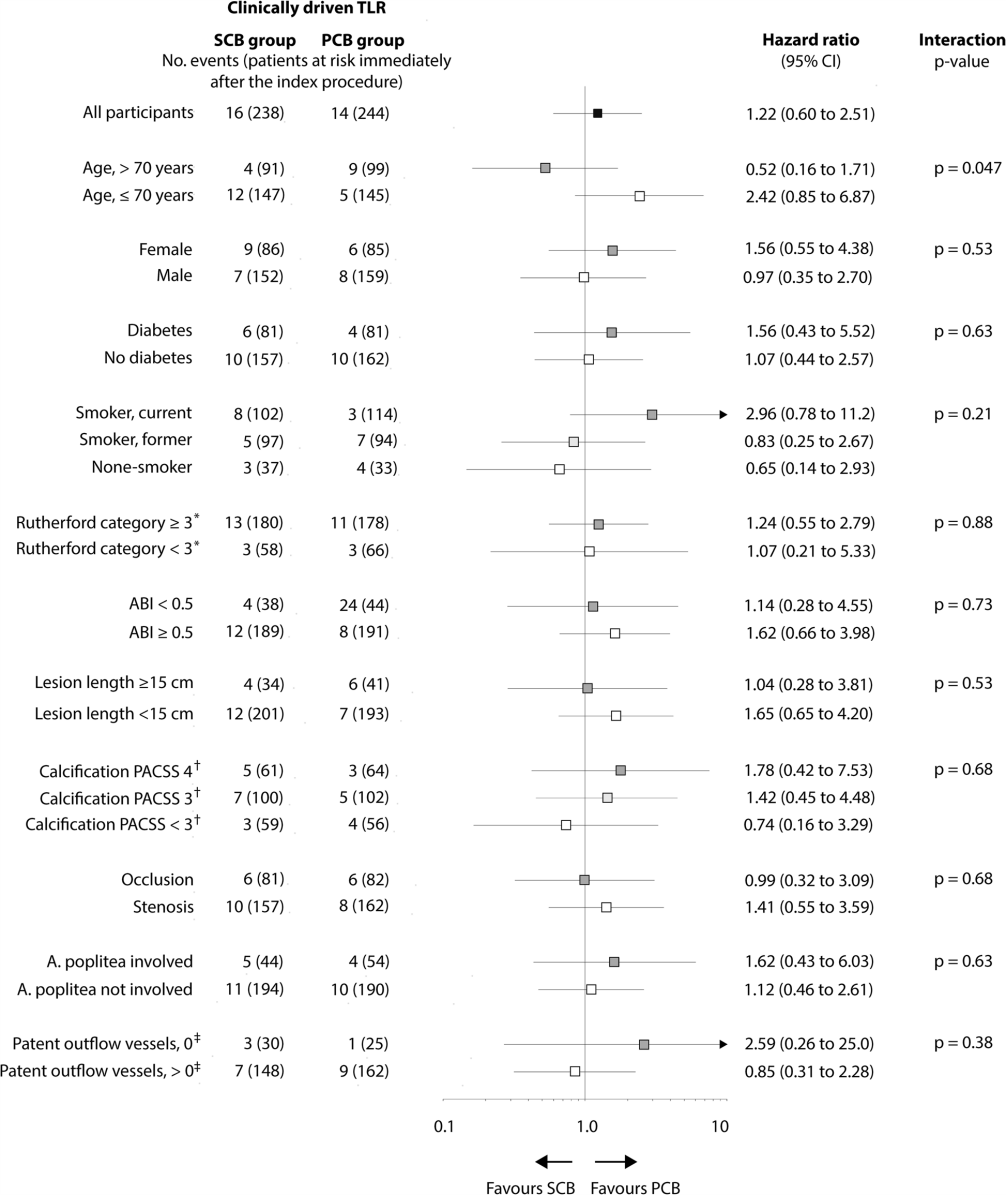
Subgroup analysis of the treatment effect of SCB versus PCB regarding clinically driven target lesion revascularisation over a period of 12 months. *Rutherford category 3 denotes moderate claudication †Chronic limb-threatening ischaemia (Rutherford category > 3) occurred only in a few participants (7 (2.9%) in the SCB group and 10 (4.1%) in the PCB group). ‡ PACSS grade 3 denotes bilateral calcification < 5 cm and PACSS grade 4 bilateral calcification ≥ 5 cm. § Patency of outflow vessels was defined as < 50% diameter stenosis. ABI = ankle-brachial index; PACSS = peripheral arterial calcium scoring system; PCB = paclitaxel-coated balloon; TLR = target lesion revascularisation; SCB = sirolimus-coated balloon

A total of 203 (85.3%) and 199 (81.6%) of the participants with available-case primary patency assessment at 12 months were included in the subgroup and post-baseline factor analyses of primary patency, respectively. The analyses of the risk (HR) of cdTLR included all participants at baseline (238 and 244, respectively) with deaths, withdrawals, and loss to follow-up being censored throughout 12 months. Counts of participants and events per subgroup are shown in Figs. 1 and 2.

### Subgroups

No treatment effect of SCB compared to PCB was observed in the entire study group in terms of 12-month primary patency adjusted for centre effects (OR 0.99 [95% CI 0.59 to 1.68]). This finding was consistent across all subgroups, with minor variations (Fig. 1). There was also no significant difference in the risk of cdTLR between the two groups within the whole study population (HR 1.22 [95% CI 0.60 to 2.51]). Except for age, no analysed subgroup suggested an interaction with cdTLR. In participants aged over 70 years, a lower risk of cdTLR was observed with SCB compared to PCB, whereas in participants aged 70 years or under, the risk increased with SCB (HR 0.52 [> 70 years] and HR 2.42 [≤ 70 years]; interaction *p*-value 0.047; Fig. 2). However, this finding was directionally inconsistent with the related outcome of primary patency (OR 0.78 [> 70 years] and OR 1.08 [≤ 70 years]; interaction *p*-value 0.41; Fig. 1), limiting its interpretability. Comparing the baseline characteristics of participants by age (≤ 70 years vs. > 70 years), it was found that those over 70 were more likely to be female and current smokers, and to have CAD and renal insufficiency. Their lesions were longer and more frequently heavily calcified, as well as involving the popliteal artery more often. However, the proportion of CTOs did not differ considerably between the age groups, and the mean CTO length was significantly shorter in the elderly. Nevertheless, adjunctive treatment, including bailout stenting, was performed less frequently among older participants. The proportion of participants with a residual diameter stenosis > 30% did not differ between age groups. Baseline characteristics between the SCB and PCB groups by age were well-balanced, however in younger participants, PCB was associated with significantly more non-flow-limiting dissections (Table 1).

**Table 1:** Participant-, lesion, and procedural characteristics according to age by treatment group

	Age ≤ 70 years			Age > 70 years			Age ≤ 70 years vs > 70 years
	SCB	PCB	*p*-value	SCB	PCB	*p*-value	*p*-value
	*n* = 147	*n* = 145		*n* = 91	*n* = 99		
Participant characteristics							
Female sex	42 (29)	37 (26)	*p* = 0.65	44 (48)	48 (48)	*p* = 0.90	*p* < 0.001
Current smoker	83 (56)	93/144 (65)	*p* = 0.16	19/89 (21)	21/97 (22)	*p* > 0.99	*p* < 0.001
Comorbidities							
Diabetes mellitus	49(33)	46/144 (32)	*p* = 0.87	32 (35)	35 (35)	*p* = 0.90	*p* = 0.62
Hypertension	128 (87)	120(83)	*p* = 0.39	81 (89)	89 (90)	*p* = 0.97	*p* = 0.15
Hyperlipidaemia	109 (74)	115 (79)	*p* = 0.37	72 (79)	80 (81)	*p* = 0.91	*p* = 0.39
CAD	39 (26)	40 (28)	*p* = 0.94	32 (35)	45 (45)	*p* = 0.20	*p* = 0.002
Renal insufficiency	19 (13)	19 (13)	*p* = 0.90	22 (24)	26 (26)	*p* = 0.87	*p* < 0.001
Med. history of MI	23 (16)	25 (17)	*p* = 0.83	14 (15)	18 (18)	*p* = 0.70	*p* = 0.91
Med. history of CVD	11/145 (8)	8/144 (6)	*p* = 0.65	7/88 (8)	11 (11)	*p* = 0.62	*p* = 0.23
Rutherford category ≥ 3	110 (75)	103 (71)	*p* = 0.55	70 (77)	75 (76)	*p* = 0.99	*p* = 0.41
CLTI (Rutherford category > 3)	3 (2)	5 (3)	*p* = 0.50	4 (4)	5 (5)	p > 0.99	*p* = 0.31
ABI < 0.5	25/145 (17)	21/142 (15)	*p* = 0.69	13/82 (16)	23/93 (25)	*p* = 0.19	*p* = 0.27
Lesion characteristics*							
Lesion length, mm	80 ± 59	77 ± 57	*p* = 0.66	91 ± 65	95 ± 63	*p* = 0.70	*p* = 0.01
Long lesions (≥ 15 cm)	18/145 (12)	21/140 (15)	*p* = 0.64	16/90 (18)	20/94 (21)	*p* = 0.58	*p* = 0.12
Total occlusion	53 (36)	47 (32)	*p* = 0.60	28 (31)	34/98 (35)	*p* = 0.68	*p* = 0.74
Occlusion length, mm	58 ± 47	62 ± 44	*p* = 0.68	35 ± 34	43 ± 45	*p* = 0.48	*p* < 0.001
Calcification PACSS Grade 4	33/135 (24)	27/130 (21)	*p* = 0.57	31/88 (35)	34/89 (38)	*p* = 0.80	*p* = 0.001
Popliteal artery involved	19 (13)	20 (14)	*p* = 0.87	25 (27)	32 (32)	*p* = 0.57	*p* < 0.001
Procedural data							
No. of DCB > 1	32 (22)	29 (20)	*p* = 0.82	23 (25)	34 (34)	*p* = 0.25	*p* = 0.03
Older-Generation PCB* used	–	31 (21)	–	–	17 (17)	–	*p* = 0.52
SCB used instead of PCB	–	1 (1)	–	–	4 (4)	–	*p* = 0.07
DCB dilation time, min	2.8 ± 0.7	2.7 ± 0.6	*p* = 0.02	2.8 ± 0.6	2.8 ± 0.4	*p* = 0.53	*p* = 0.30
Dissection NHLBI Type A–C^†^	45/143 (31)	65/136 (48)	*p* = 0.008	41/85 (48)	47/95 (49)	*p* = 0.99	*p* = 0.046
Dissection NHLBI Type D or E^†^	61/143 (43)	50/136 (37)	*p* = 0.38	32/85 (38)	34/95 (36)	*p* = 0.92	*p* = 0.50
Adjunctive treatment^‡^	65 (44)	68 (47)	*p* = 0.73	27 (30)	39 (39)	*p* = 0.91	*p* = 0.02
Bailout stenting^†^	32 (22)	28 (19)	*p* = 0.71	11 (12)	10/98 (10)	*p* = 0.82	*p* = 0.01
Residual stenosis > 30%^§^	43/145 (30)	55/144 (38)	*p* = 0.16	31 (34)	36/97 (37)	*p* = 0.78	*p* = 0.70

Data n/N (%) or mean (SD)

^*^Lutonix™ (Becton, Dickinson and Company, Franklin Lakes, NJ, USA): 2.0 μg paclitaxel/mm^2^, polysorbate and sorbitol excipient

^†^Adjudicated and reported by the core laboratory

^‡^Adjunctive treatment of the target lesion (postdilation with the used DCB or standard balloon and/or bailout stenting with bare-metal stents)

^§^Residual diameter stenosis after all treatment, including any adjunctive treatment where applicable

*CAD* coronary artery disease, *CLTI* chronic limb-threatening ischaemia, *CVD* cerebrovascular disease, *MI* myocardial infarction, *NHLBI grading system* National Heart, Lung, and Blood Institute grading system, *PACSS* peripheral arterial calcium scoring system, *PCB* paclitaxel-coated balloon, *SCB* sirolimus-coated balloon, *SFA* superficial femoral artery

### Post-baseline factors

**Fig. 3:**
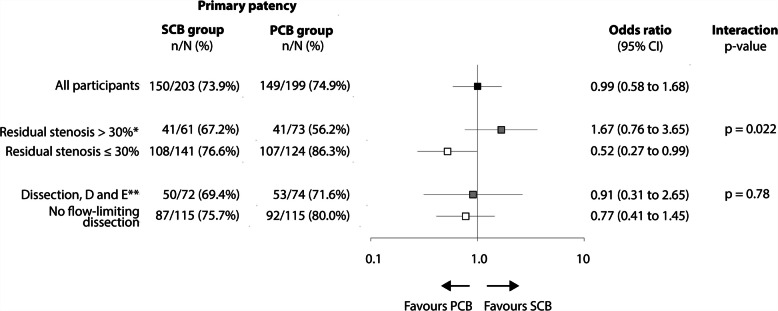
Influence of post-baseline factors on the treatment effect of SCB versus PCB on primary patency at 12 months, controlling for centre effects. *Residual diameter stenosis (after all treatment, including any adjunctive treatment where applicable) was assessed by the core laboratory. **Dissections were graded according to the National Heart, Lung, and Blood Institute (NHLBI) system, in which grades D and E denote delayed but complete distal flow and delayed antegrade flow, respectively. Dissection grades were adjudicated by the core laboratory. PCB = paclitaxel-coated balloon; SCB = sirolimus-coated balloon

**Fig. 4:**
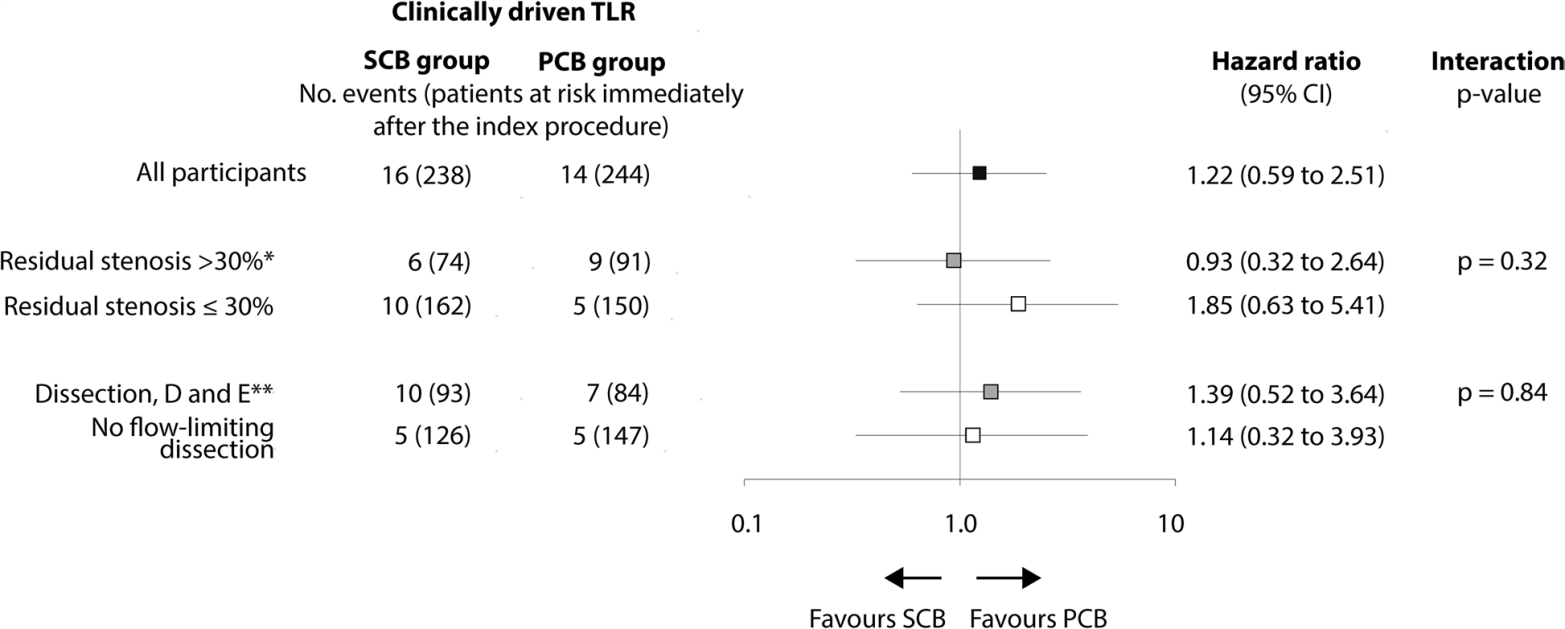
Influence of post-baseline factors on the treatment effect of SCB versus PCB on clinically driven TLR over a period of 12 months. *Residual diameter stenosis (after all treatment, including any adjunctive treatment where applicable) was assessed by the core laboratory. **Dissections were graded according to the National Heart, Lung, and Blood Institute (NHLBI) system, in which grades D and E denote delayed but complete distal flow and delayed antegrade flow, respectively. Dissection grades were adjudicated by the core laboratory. PCB = paclitaxel-coated balloon; SCB = sirolimus-coated balloon, TLR = target lesion revascularisation

Residual stenosis > 30% was associated with better outcomes after SCB, whereas ≤ 30% residual stenosis was associated with better outcomes after PCB. This is reflected in primary patency ORs of 1.67 and 0.52 and an interaction *p*-value of 0.022 (Fig. 3). Although the effect of SCB versus PCB on cdTLR was directionally consistent, its magnitude was insufficient to demonstrate a meaningful interaction (Fig. 4). As shown in Table 2, participants with a residual diameter stenosis of > 30% of both treatment groups were more likely to have longer lesions, chronic total occlusions, and heavily calcified lesions. Flow-limiting dissections were more frequent, and an older-generation PCB type (Lutonix™) was used more frequently in lesions in which a residual diameter stenosis of > 30% occurred (42% vs. 5% in lesions with ≤ 30% residual stenosis, *p* < 0.001). Participant and lesion characteristics according to residual diameter stenosis were balanced between groups. However, SCBs were dilated longer than PCBs in lesions with residual diameter stenosis > 30% (3.0 ± 0.8 min vs. 2.7 ± 0.6 min, *p* = 0.004).

**Table 2:** Participant-, lesion, and procedural characteristics according to residual diameter stenosis by treatment group

	Residual diameter stenosis ≤ 30%			Residual diameter stenosis > 30%			DS ≤ 30% vs. > 30%
	SCB	PCB	*p*-value	SCB	PCB	*p*-value	*p*-value
	*n* = 162	*n* = 150		*n* = 74	*n* = 91		
Participant characteristics							
Age > 70 years	60 (37)	61 (41)	*p* = 0.51	31 (42)	36 (40)	*p* = 0.89	*p* = 0.70
Female sex	59 (36)	54 (36)	*p* = 0.97	27(36)	29 (32)	*p* = 0.65	*p* = 0.69
Current smoker	71/160 (44)	74/148 (50)	*p* = 0.32	30 (41)	39/90 (43)	*p* = 0.84	*p* = 0.30
Comorbidities							
Diabetes mellitus	53 (33)	46/149 (31)	*p* = 0.82	27 (36)	33 (36)	*p* = 0.89	*p* = 0.32
Hypertension	142 (88)	130 (87)	*p* = 0.93	65 (88)	77 (85)	*p* = 0.65	*p* = 0.84
Hyperlipidaemia	126 (78)	121(81)	*p* = 0.63	54 (73)	71 (78)	*p* = 0.57	*p* = 0.46
CAD	46 (28)	54 (36)	*p* = 0.19	26 (35)	30 (33)	*p* = 0.90	*p* = 0.75
Renal insufficiency	29 (18)	25 (17)	*p* = 0.89	12 (16)	20 (22)	*p* = 0.43	*p* = 0.66
Med. history of MI	24 (15)	29 (19)	*p* = 0.36	13 (18)	14 (15)	*p* = 0.83	*p* = 0.96
Med. history of CVD	8 (5)	4 (3)	*p* = 0.38	7 (9)	11 (12)	*p* = 0.63	*p* = 0.005
Rutherford category ≥ 3	120 (74)	114 (76)	*p* = 0.79	58 (78)	63 (69)	*p* = 0.18	*p* = 0.77
CLTI (Rutherford category > 3)	5 (3)	7 (5)	*p* = 0.56	2 (3)	3 (3)	*p* > 0.99	*p* = 0.30
ABI < 0.5	27/154 (18)	26/143 (18)	*p* > 0.99	10/71 (14)	18/89 (20)	*p* = 0.31	*p* = 0.97
Lesion characteristics*							
Lesion length, mm	71 (55)	74 (58)	*p* = 0.47	115 (66)	101 (61)	*p* = 0.43	*p* < 0.001
Long lesions (≥ 15 cm)	17/160 (11)	17/146 (12)	*p* = 0.86	18/73 (25)	24/88 (27)	*p* = 0.71	*p* < 0.001
Total occlusion	50 (31)	44 (29)	*p* = 0.77	30 (41)	36 (40)	*p* = 0.98	*p* = 0.03
Occlusion length, mm	46 (36)	49 (48)	*p* = 0.18	48 (43)	48 (38)	*p* = 0.17	*p* = 0.15
Calcification PACSS Grade 4	35/152 (23)	35/134 (26)	*p* = 0.64	29/70 (41)	26/84 (31)	*p* = 0.24	*p* = 0.02
Popliteal artery involved	29 (18)	37 (25)	*p* = 0.19	18 (24)	18 (20)	*p* = 0.57	*p* = 0.96
Procedural data							
No. of DCB > 1	25 (15)	24 (16)	*p* = 0.99	29 (39)	37 (41)	*p* = 0.98	*p* < 0.001
Older generation PCB* used	–	7 (5)	–	–	38 (42)	–	*p* < 0.001
SCB used instead of PCB	–	2 (1)	–	–	3 (3)	–	*p* = 0.57
DCB dilation time, min	2.8 (0.6)	2.8 (0.5)	*p* > 0.99	3.0 (0.8)	2.7 (0.6)	*p* = 0.004	*p* = 0.47
Dissection NHLBI Type A–C^†^	65/153 (42)	78/144 (54)	*p* = 0.044	21/66 (32)	34/86 (40)	*p* = 0.42	*p* = 0.02
Dissection NHLBI Type D or E^†^	52/153 (34)	38/144 (26)	*p* = 0.19	41/66 (62)	45/86 (52)	*p* = 0.30	*p* < 0.001
Adjunctive treatment^‡^	50 (31)	46 (31)	*p* = 0.93	16 (22)	27 (30)	*p* = 0.24	*p* = 0.33
Bailout stenting^†^	45 (28)	33 (22)	*p* = 0.30	11 (15)	17 (19)	*p* = 0.51	*p* = 0.06

Data are *n*/*N* (%) or mean (SD)

^*^Lutonix™ (Becton, Dickinson and Company, Franklin Lakes, NJ, USA): 2.0 μg paclitaxel/mm^2^, polysorbate and sorbitol excipient

^†^Adjudicated and reported by the core laboratory

^‡^Adjunctive treatment of the target lesion (post-dilation with the used DCB or standard balloon and/or bailout stenting with bare-metal stents)

*CAD* coronary artery disease, *CLTI* chronic limb-threatening ischaemia, *CVD* cerebrovascular disease, *DS* diameter stenosis, *MI* myocardial infarction, *NHLBI grading system* National Heart, Lung, and Blood Institute grading system, *PACSS* peripheral arterial calcium scoring system, *PCB* paclitaxel-coated balloon, *SCB* sirolimus-coated balloon, *SFA* superficial femoral artery

## Discussion

The underlying randomised SIRONA trial revealed non-inferiority in primary patency with SCB compared to PCB in patients with femoropopliteal artery disease categorised as Rutherford 2 to 4 at 12 months. However, this result was achieved with a narrow margin and was not robust in sensitivity analysis. Therefore, this exploratory post-hoc analysis evaluated whether treatment effects differed across subgroups or relevant modifying post-baseline factors. The evaluation showed consistency among subgroups. Two nominal treatment interactions were observed. However, these findings should be interpreted with considerable caution because numerous interaction tests were performed and event numbers were limited. Accordingly, these observations should be considered hypothesis-generating only.

The observed age interaction for cdTLR may represent a chance finding, especially since interaction with age was based on low event rates and was not reflected in primary patency, reducing confidence in this finding. However, as there was no age-related advantage for SCB in terms of baseline and procedural characteristics, one might hypothesise that vessel wall conditions in elderly participants provide a more favourable basis for the properties of sirolimus. These include its strong anti-inflammatory effects and regulation of cell proliferation in hypoxic microenvironments, particularly within the deeper media layers [9, 10]. In mouse models, ageing exacerbates the formation of neointima in response to mechanical injury. However, vascular smooth muscle cells from ageing mice are less susceptible to apoptosis [13]. It remains to be clarified whether this is also true for humans and whether it reduces apoptotic properties of paclitaxel in elderly patients. Another possible association between age and cdTLR is the proportion of non-flow-limiting dissections. A previous study on coronary arteries showed that late lumen enlargement (LLE) following DCB angioplasty, probably resulting from vessel enlargement and plaque regression, was strongly associated with non-flow limiting dissections [14, 15]. Yamamoto et al. discussed that paclitaxel diffuses only slowly into the deeper layers of the media due to its hydrophobic nature and strong unspecific binding to arterial tissue proteins [14, 16]. They suggested that dissections enhance its transmural diffusion. Our subgroup analysis supported this, showing that a higher proportion of non-flow limiting dissections in younger participants who received PCB was associated with a lower risk of TLR. Although older participants had slightly more non-flow-limiting dissections than the younger participants, there was no difference between the treatment groups. A recent meta-analysis revealed that LLE occurred in 50% of patients following coronary PCB and in 28% following coronary SCB [7]. The authors of this meta-analysis attributed this finding to the higher lipophilicity of paclitaxel. However, other drug-specific properties, such as binding to tissue proteins, may also be important. Unlike paclitaxel, which remains mainly subintimal and accumulates in the adventitia, sirolimus distributes more evenly within the vascular layers [8]. Further investigation is needed to determine whether the effects of sirolimus and paclitaxel depend on vascular ageing.

A nominal interaction between treatment and residual diameter stenosis was observed for primary patency. Although the estimated treatment effect favoured SCB in lesions with residual diameter stenosis > 30%, it remains unclear whether this observation reflects a true biological difference or a chance finding. These lesions were more complex at baseline in terms of length, severe calcification, and CTO, with a subsequently higher proportion of flow-limiting dissections, but similar proportion of non-flow-limiting dissections. Nevertheless, bailout stenting was performed somewhat less frequently. Despite the lower rate of adjunctive treatment including bailout stenting with SCB, even not significantly different to PCB, SCB achieved a better primary patency rate than PCB. It remains to be evaluated whether this was due to chance or to specific distribution properties of sirolimus, or whether the significantly longer dilation time with SCB compared to PCB in these lesions contributed to the favourable results. Finally, the benefits of SCB in case of a higher percentage of residual stenosis may also be due to the weakness of the control group in this cohort, which included a significantly high proportion of older-generation PCB types with a comparably low efficacy [1]. Conversely, more non-flow-limiting dissections occurred with PCB in lesions that resulted in ≤ 30% residual stenosis. This may have led to a higher rate of LLE [14, 15] and, subsequently, to a higher rate of primary patency.

### Study limitations

Firstly, our study was a post hoc subgroup analysis without stratification before randomisation. Therefore, the balance of confounders from randomisation may not have been preserved within subgroups. For example, this occurred when comparing the SCB and PCB dilation times of lesions resulting in over 30% residual diameter stenosis. Secondly, there are relatively few cdTLR events with which to assess subgroups. However, as cdTLR corresponds to primary patency in a clinical context, it may be useful for more accurately assessing the credibility of the results when comparing them in terms of both outcomes [17]. For instance, the results for cdTLR and primary patency do not confirm each other regarding treatment interaction with age, which limits the credibility of these findings. Furthermore, post-baseline factors themselves may be affected by the treatment. Thirdly, numerous subgroup interaction tests were performed without adjusting for multiple comparisons. Consequently, nominal interaction *p*-values may represent chance findings and should not be interpreted as confirmatory evidence. Finally, the investigators used different types of PCB with considerably different levels of effectiveness as the control group, which may have affected the results of our head-to-head comparison. Therefore, our investigation should be considered exploratory, but it may highlight the need for further study.

## Conclusions

The similar effectiveness of SCB and PCB angioplasty in patients with femoropopliteal artery lesions was confirmed by mainly consistent results across subgroups. However, it remains to be demonstrated whether the apparent benefit in favour of SCB for patients older than 70 years reflects specific properties of sirolimus.

## Data Availability

The data that support the findings of this study are not publicly available due to study participant privacy restrictions and are available from the corresponding author on reasonable request.

## Abbreviations

CAD: Coronary artery disease
CdTLR: Clinically driven target lesion revascularisation
CTO: Chronic total occlusion
LLE: Late lumen enlargement
PCB: Paclitaxel-coated balloon
SCB: Sirolimus-coated balloon
TVR: Target vessel revascularisation
